# Lack of Association between Polymorphisms of the *TLR4* Gene and Infection with the Hepatitis B and C Viruses

**DOI:** 10.1155/2015/150673

**Published:** 2015-08-06

**Authors:** Orlando de Souza Pires-Neto, Keyla Santos Guedes de Sá, Barbara Brasil Santana, Samara Tatielle Monteiro Gomes, Ednelza da Silva Graça Amoras, Simone Regina da Silva Conde, Sâmia Demachki, Vânia Nakauth Azevedo, Rosimar Neris Martins-Feitosa, Marluísa de Oliveira Guimarães Ishak, Ricardo Ishak, Antonio Carlos Rosário Vallinoto

**Affiliations:** ^1^Laboratorio de Virologia, Instituto de Ciências Biológicas, Universidade Federal do Pará, Guamá, 66075-110 Belém, PA, Brazil; ^2^Faculdade de Medicina, Instituto de Ciências da Saúde, Universidade Federal do Pará, Umarizal, 66050-060 Belém, PA, Brazil

## Abstract

Toll-like receptor 4 (TLR4) plays a crucial role in the early recognition of pathogenic microorganisms and provides an ideal model to investigate the consequences of genetic variation and susceptibility to diseases. The present study investigated the occurrence of the single nucleotide polymorphisms (SNPs) rs4986790 (A>G) and rs4986791 (C>T) in the *TLR4* gene in chronic carriers of the hepatitis B (HBV) and C (HCV) viruses. A total of 420 blood samples were collected (HBV, 49; HCV, 72; and controls, 299) at the liver disease outpatient clinic of Hospital da Fundação Santa Casa de Misericórdia do Pará (FSCMPA). Genomic DNA extracted from leukocytes was subjected to real-time polymerase chain reaction (qPCR) analysis to identify the genetic profile of the participants. No significant differences were found in the allele and genotype frequencies between the infected participants and controls. No significant associations were found between the investigated polymorphisms and inflammatory activity, fibrosis, and the presence of cirrhosis; the same results were obtained in the haplotype analysis. The results showed a lack of association between the rs4986790 and rs4986791 SNPs and susceptibility to infection with HBV and HCV, as well as clinical and laboratory information of the patients.

## 1. Introduction

The chronic liver diseases caused by* hepatitis B virus* (HBV) and* hepatitis C virus* (HCV) are a public health concern [1, 2]. Genetic and immune differences among individuals might be involved in the mechanisms that lead to the perpetuation of these viruses in the liver, as the elimination of the viruses requires an adequate immune response [3].

Toll-like receptor 4 (TLR4) recognizes pathogen-associated molecular patterns (PAMPs). It participates in the innate immune response via activation of cell signaling pathways [4] that lead to the transcription of proinflammatory cytokine genes, such as interleukin- (IL-) 12, IL-6, tumor necrosis factor (TNF) *α*, and type I interferon (IFN) [5]. As a result, TLR4 is involved in the pathogenesis of various viral infections [6]. Therefore, TLR4 provides an ideal model to investigate the consequences of genetic variation and its relationship with receptor function and, consequently, its impact on the susceptibility to infectious diseases [7].

Polymorphisms of the* TLR4* gene are associated with delayed progression of liver fibrosis [8] and reduced risk of development of hepatocellular carcinoma [9]. The rs4986790 (A>G) and rs4986791 (C>T) SNPs of the* TLR4* gene are located in the fourth exon and affect the extracellular domain of the molecule, which is associated with receptor hyporesponsiveness [10].

TLR4 signaling results in the activation of intracellular pathways including MAPK and PI-3K/Akt in hepatocytes and reduces HBV replication in an IFN-independent manner [11]. HBV is capable of neutralizing the actions of both TLR3 and TLR2/4 through downregulation of TLR expression and the attenuation of cellular signaling pathways. Furthermore, an elevated expression of TLR2/4 on DC cell surfaces is observed in the peripheral blood synergistically, which promotes the disease progression of chronic HBV infection [12].

Uraki et al. [13] suggest a close association between the production of IL-6 and TLR4 activation in HCV infected cells. Amino acid (aa) substitution at position 70 from arginine (70R) to glutamine (70Q) at position 70 of the extracellular HCV core protein enhanced IL-6 production. This was impaired by TLR4 inhibition in about 40%, which leads to the development of hepatocellular carcinoma. Additionally, TLR4 and IL-6 serum levels are associated with the characteristic haemodynamic derangement observed in advanced phases of cirrhosis. HIV/HCV coinfected cirrhotic patients present inflammatory and systemic haemodynamic alterations similar to those observed in HCV monoinfected patients, suggesting a role of bacterial translocation [14].

Furthermore, another indirect evidence for the role of TLR4 in HCV infection was provided by Sadik et al. [15] who suggest that interleukin-10 (IL-10) is one of the upstream regulators of TLR4. A possible interplay between TLR4 and IL-10 was suggested by interplay of single-nucleotide polymorphisms (SNPs) in TLR4 and IL-10-1082 and the expression levels of these proteins in predicting the response to treatment in chronic HCV patients.

Although until now it is not completely clear how HBV or HCV induces TLR4 activation, both infections require strong initial immune response, mainly mediated by IFN *α*/*β*, which are products of activation of TLR4 [5]; it is assumed that a functional impairment due to changing in the receptor, caused by polymorphisms studied herein, may be associated with progression to chronic form of the infection and consequently the worsening of the patient's clinical and laboratory markers.

To assess the association between the rs4986790 (A>G) and rs4986791 (C>T) polymorphisms and liver infection with HBV and HCV, the present study investigated, for the first time in the Brazilian Amazonia, the prevalence of these polymorphisms in chronic infection carriers and in seronegative controls residing in the city of Belém, the capital of the state of Pará, in Northern Brazil.

## 2. Materials and Methods

### 2.1. Study Population

Samples were collected from 49 chronic HBV carriers and 72 chronic HCV carriers at the liver disease outpatient clinic of Holy House of Mercy of Pará Foundation Hospital (Hospital da Fundação Santa Casa de Misericórdia do Pará (FSCMPA)). Individuals from both genders, aged 22 to 80 years, participated in the study. A group comprised of 299 individuals of both genders who were seronegative for both HBV and HCV served as the control group.

Individuals aged 18 years or older, from both genders, who were carriers of HBsAgs for more than six months, positive for HCV-RNA, and being with or without persistently elevated alanine aminotransferase levels were included in the study. Individuals who did not meet those criteria or who were coinfected with the hepatitis D virus (HDV) and/or the human immunodeficiency virus (HIV) and patients who had used or were using specific antiviral therapy against the HBV or HCV were excluded from the study.

The patients were also evaluated by clinical and laboratory parameters, including biochemical (alanine aminotransferase (ALT), aspartate aminotransferase (AST), gamma-glutamyl transferase (GGT), and prothrombin time (PT)) and serological tests (HBsAg, HBeAg, anti-HBeAg, anti-HBc total, and anti-HCV) and histopathology of liver biopsy.

All eligible individuals were informed of the study aims, and those who agreed to voluntary participation signed an informed consent form. The study was approved by the research ethics committee of Federal University of Pará, protocol number 684,432/2014, in compliance with National Health Council Resolution number 466/2012, which contains regulatory norms and guidelines for research involving human beings.

### 2.2. Sample Collection and Histopathological Analysis

Blood samples were collected in vacuum tubes containing the anticoagulant ethylenediaminetetraacetic acid (EDTA); the plasma was separated by centrifugation and stored at −20°C until analysis. An ultrasound-guided biopsy was performed with a Tru-Cut needle. The liver specimens were separated into two parts. One part was examined at the Department of Anatomical Pathology, Federal University of Pará (Universidade Federal do Pará (UFPA)), according to standard protocol, and stained with the hematoxylin-eosin (HE), chromotrope-aniline blue (CAB), Gomori's reticulin, and Shikata orcein methods. A histopathological diagnosis was made based on the French METAVIR classification (METAVIR Cooperative Study Group); the activity of portal and periportal inflammatory infiltrates was scored from 0 to 3, and structural abnormalities were scored from 0 to 4. The other part of the biopsy specimen was sent to the Laboratory of Virology, Institute of Biological Sciences/UFPA (Instituto de Ciências Biológicas/Universidade Federal do Pará (ICB/UFPA)), for genetic investigation and stored at −70°C until analysis.

### 2.3. Genetic Analysis

DNA was extracted from peripheral blood leukocytes according to the manufacturer's instructions (Gentra Systems, Inc., Minneapolis, MN, USA). The procedure included cell lysis, protein precipitation, DNA precipitation, and DNA hydration.

The extracted DNA was subjected to real-time quantitative polymerase chain reaction (qPCR) assays using the Step One Plus Sequence Detector (Life Technologies, Foster City, CA, USA). The assays used for each polymorphism included a primer pair and a pair of probes; VIC and FAM labeling was performed for each allele of the respective polymorphisms. Each reaction included 3.5 *μ*L H_2_O, 5.0 *μ*L TaqMan Universal PCR Master Mix [2X], 0.5 *μ*L TaqMan Assay [20X], and 1 *μ*L DNA, with a final volume of 10 *μ*L. The following cycling and temperature protocol was used for amplification and detection of alleles: 60°C for 30 seconds, 95°C for 10 minutes, and 50 cycles at 92°C for 30 seconds and 60°C for 90 seconds. The C_11722238_20 and C_11722237_20 assays (Life Technologies, Foster City, CA, USA) were used for the rs4986790 and rs4986791 polymorphisms, respectively.

### 2.4. Statistical Analysis

The genotype and allele frequencies were calculated based on direct counting. The Hardy-Weinberg equilibrium was assessed with a chi-square test. The allele, genotype, and haplotype frequencies were compared among the groups using Fisher's exact test and a* G*-test. The association between genetic models (dominant, recessive, and overdominant) and the risks of HBV and HCV infection were analyzed with simple logistic regression. The risk of progression of the disease was calculated as an odds ratio (OR) with a 95% confidence interval. Statistical analyses were performed using the Bioestat 5.3 software; the significance level was set as *p* < 0.05.

## 3. Results

The population data and measurements of biochemical markers are summarized in Table 1. ALT (82.77 IU/L), AST (72.21 IU/L), and GGT (87.49 IU/L) mean levels were higher in the group with HCV, but the HBV group presented the lowest prothrombin time (78.27%). Correlation analysis of biochemical data of liver function in the presence of the studied polymorphisms did not show significant results in patients infected with HCV. The analysis was not done for HBV infected subject as only one patient was studied.

Samples from 49 chronic HBV carriers, 72 chronic HCV carriers, and 299 controls were genotyped. All three groups met the Hardy-Weinberg equilibrium assumptions. Cirrhosis was exhibited by nine of the 49 participants infected with HBV and 17 of the 72 participants infected with HCV.

For the rs4986790 polymorphism, the wild-type homozygous AA genotype was most frequently observed among the participants with hepatitis B (97.96%), hepatitis C (93.06%), and control (92.31%). For the rs4986791 polymorphism, the wild-type homozygous CC genotype was most frequently observed among the participants with hepatitis B (97.56%), hepatitis C (94.44%), and control (92.31%). The frequency of either SNP did not show a significant difference compared to the control group (Table 2).

Six haplotypes were identified in the patient and control groups, and the AA/CC haplotype was most frequently observed in all three groups (HBV = 95.12%, HCV = 93.05%, and controls = 90.64%). No association was observed between the haplotype and viral infection.

No significant result was found in the comparison of polymorphisms with inflammatory activity, degree of fibrosis, and cirrhosis (Table 3). None of the genetic models of dominance, recessivity, or overdominance exhibited an association with risk of infection with HBV (Table 4) or HCV (Table 5).

## 4. Discussion

TRL4 is a transmembrane receptor and one of the main Toll-like receptors (TLRs). It is comprised of a leucine-rich extracellular domain responsible for recognition, a transmembrane domain, and an intracellular domain that is similar to the IL-1 receptor [16]. The molecule promotes the innate immune response through activation of TIR-domain-containing adapter-inducing interferon-*β* (TRIF) and myeloid differentiation primary response gene 88 (MYD88) adapters [17], which induce production of proinflammatory cytokines.

Two polymorphisms of TLR4 rs4986790 (A>G) and rs4986791 (C>T) are well known. These polymorphisms lead to conformational changes that might interfere with the interaction between the receptor and ligand and with protein stability [18], in addition to causing deficient recruitment of MYD88 and TRIF, but they have no effect on receptor expression [19].

TLR4 plays a crucial role in the promotion of IFN *α*/*β* transcription, which directs the response that targets products of viral replication [3], because the liver is an organ rich in innate immune response cells, such as nature killer (NK) and Kupffer cells [20]. Previous studies have also shown a relationship between TLR4 and infection with HBV and HCV, indicating that HCV NS5A protein induces the expression of TLR4 [6] and that TLR4 is regulated by HBV in the hepatocytes of chronic carriers of the virus [21], resulting in inhibition of viral replication through increased production of IFNs [22]. Additionally, recent evidences show the association of TLR4 with HBV and HCV infections by the activation of intracellular pathways including MAPK and PI-3K/Akt and IL-6 and IL-10 signaling [11, 13, 15]. TLR4 and IL-6 serum values have been associated with the characteristic haemodynamic derangement of HIV/HCV coinfected cirrhotic patients who present inflammatory and systemic haemodynamic alterations, the same observed in HCV monoinfected patients, suggesting a role of bacterial translocation [14].

The liver injury caused by HBV and HCV is mainly mediated by the host's immune response to the viral proteins [23]. The SNPs investigated in the present study are associated with a different pattern of cytokine production in individuals with cirrhosis [24], which might contribute to the development of clinical complications. However, the present study showed no association between these polymorphisms and inflammatory activity, the degree of fibrosis, or cirrhosis. Therefore, the change in TLR4 induced by these polymorphisms does not seem to modify the course of the infection with HBV and HCV with regard to the TLR4-mediated immune response, at least in the groups analyzed in the present study.

Controversial results have been reported regarding the association between the SNPs investigated in the present study and HBV and HCV infection. In the present study, no significant association was observed regarding the susceptibility to infection and inflammatory activity, the degree of fibrosis, cirrhosis, or disease progression. These findings corroborate those reported by Nakamura et al. [25], in Japan, who studied 260 patients, without detecting any polymorphic allele for both SNPs, and those reported by Agúndez et al. [26] in a Spanish population who also found a low frequency of polymorphic allele of the SNP rs4986791 in both groups of 153 infected persons (CC = 85.6% = 13.7% CT and TT = 0.7%) and 390 controls (CC = 87.5%, 12.1% and CT = TT = 0.4%). However, in a study conducted in a population of 450 Saudis, Al-Qahtani et al. [27] found a significant association between both SNPs and HCV infection, but not with disease progression. This can be explained by the high frequency of polymorphisms in the population studied, as can be seen in allele frequencies of the control population (G = 10.6% and T = 11.0%). Cussigh et al. [28] found that, in Caucasian populations, the rs4986790 polymorphism AA genotype might influence the progression of liver fibrosis in chronic HBV carriers.

The frequencies of polymorphic alleles vary among different populations. In our study, the frequencies of the investigated SNPs varied from 2 to 7% in all of the study groups. These SNPs are rare in Chinese [29] and Thai populations [30], while they appear in 10% of Caucasian and African populations [31]. Studies conducted with other Brazilian samples have reported frequencies of heterozygosis similar to ours [32–34]. These findings suggest the heterogeneous distribution of the frequencies of the investigated SNPs among populations of different ethnic origins. In addition, such a differential distribution might contribute to the well-established variation in the response to pathogenic microorganisms among individuals from different ethnic groups [35, 36]. The three groups analyzed in the present study were selected from the overall population in Belém, the capital of Pará, which is characterized by the miscegenation of the main three ethnic groups that formed the Brazilian population: white colonizers, Native American Indians, and black African individuals brought to Brazil as slaves between the 16th and 18th centuries.

The results of the present study revealed the low frequency of polymorphic alleles in the population and a lack of association between the rs4986790 and rs4986791 SNPs and susceptibility to HCV and HBV infection. Currently, our group is assessing the correlation of* TLR4* gene expression in hepatic tissue of the chronically infected HBV and HCV patients.

## 5. Conclusion

The present study investigated, for the first time in the Brazilian Amazonia, the prevalence of the* TLR4* gene polymorphisms in HCV and HBV chronic infection carriers and the results showed a lack of association between the rs4986790 and rs4986791 SNPs and susceptibility to both virus infections as well as the clinical and laboratory information of the patients.

## Figures and Tables

**Table 1:** Clinical and laboratory information about the HBV and HCV infected patients.

Variables	HBV chronic hepatitis	HCV chronic hepatitis
Study subjects (*n*)	39	72
Gender (female/male)	16/33	33/39
Age (years) mean ± SD	49.21 ± 15.10	52.56 ± 10.05
ALT (UI/L) mean ± SD	70.06 ± 90.89	82.77 ± 61.12
AST (UI/L) mean ± SD	58.89 ± 56.87	72.21 ± 51.35
GGT (UI/L) mean ± SD	58.98 ± 101.43	87.49 ± 75.46
PT (%) mean ± SD	78.27 ± 20.76	88.61 ± 16.56

SD: standard deviation.

**Table 2:** Genotype, allele, and haplotype frequencies of SNPs of the *TLR4* gene in the investigated groups.

SNPs	HBV *n* (%)	HCV *n* (%)	Control *n* (%)	p1	p2
rs4986790 (A>G)					
AA	48 (97.96)	67 (93.06)	276 (92.31)	0.2445	0.7990
AG	1 (2.04)	5 (6.94)	22 (7.36)		
GG	0	0	1 (0.33)		
A	97 (98.98)	139 (96.52)	574 (95.99)	0.1903	0.9510
G	1 (1.02)	5 (3.47)	24 (4.01)		
rs4986791 (C>T)					
CC	40 (97.56)	68 (94.44)	276 (92.31)	0.3641	0.6891
CT	1 (2.44)	4 (5.56)	22 (7.36)		
TT	0	0	1 (0.33)		
C	81 (98.78)	140 (97.22)	574 (95.99)	0.3009	0.6417
T	1 (1.22)	4 (2.78)	24 (4.01)		
Haplotypes					
AA/CC	39 (95.12)	67 (93.05)	271 (90.64)	0.6409	0.9305
AA/CT	1 (2.44)	0	5 (1.67)		
AA/TT	0	0	0		
AG/CC	1 (2.44)	1 (1.39)	4 (1.34)		
AG/CT	0	4 (5.56)	17 (5.69)		
AG/TT	0	0	1 (0.33)		
GG/CC	0	0	1 (0.33)		
GG/CT	0	0	0		
GG/TT	0	0	0		

p1: HBV versus control; p2: HCV versus control.

**Table 3:** Association of polymorphisms of the *TLR4* gene with inflammatory activity, degree of fibrosis, and liver cirrhosis in chronic HBV and HCV carriers.

Infection	Inflammatory activity		*p*	Degree of fibrosis		*p*	Cirrhosis		*p*
	0-1 (%)	2-3 (%)		0–2 (%)	3-4 (%)		Yes (%)	No (%)	
HBV									
rs4986790									
AA	19 (82.6)	4 (17.4)	1.000	20 (87)	03 (13)	1.000	9 (18.8)	39 (81.2)	1.000
AG/GG	01 (100)	0		01 (100)	0		0	01 (100)	
rs4986791									
CC	17 (85)	03 (15)	0.191	17 (85)	03 (15)	1.000	7 (17.5)	33 (82.5)	1.000
CT/TT	0	01 (100)		01 (100)	0		0	01 (100)	

HCV									
rs4986790									
AA	34 (64.1)	19 (35.9)	0.146	38 (70.4)	16 (29.6)	0.634	17 (25.4)	50 (74.6)	0.331
AG/GG	01 (20)	04 (80)		03 (60)	02 (40)		0	05 (100)	
rs4986791									
CC	34 (63)	20 (37)	0.290	36 (69.2)	16 (30.8)	0.587	17 (25)	51 (75)	0.567
CT/TT	01 (25)	03 (75)		02 (50)	02 (50)		0	04 (100)	

**Table 4:** Genetic models and risk of infection with HBV.

Genetic model	Genotype	Frequencies		OR	95% CI	*p*
		Control *n* (%)	HBV *n* (%)			
	rs4986790					
Dominant	AA	276 (92.3)	48 (98.9)	1	0.03–1.89	0.9574
	AG + GG	23 (7.7)	1 (2.0)	0.2500		
Recessive	AA + AG	298 (99.7)	49 (100)	1	—	1
	GG	1 (0.3)	0 (0)	0		
Overdominant	AA + GG	277 (92.6)	48 (98.0)	1	0.03–1.99	0.9577
	AG	22 (7.4)	1 (2.0)	0.2623		

	rs4986791					
Dominant	CC	276 (92.0)	40 (97.6)	1	0.04–2.28	0.9574
	CT + TT	23 (8.0)	1 (2.4)	0.3		
Recessive	CC + CT	298 (99.67)	41 (100)	1	—	1
	TT	1 (0.33)	0 (0)	0		
Overdominant	CC + TT	277 (92.64)	40 (97.6)	1	0.04–2.40	0.9577
	CT	22 (7.36)	1 (2.4)	0.3148		

**Table 5:** Genetic models and risk of infection with HCV.

Genetic model	Genotype	Frequencies		OR	95% CI	*p*
		Control *n* (%)	HCV *n* (%)			
	rs4986790					
Dominant	AA	276 (92.3)	67 (93.1)	1	0.33–2.44	0.9048
	AG + GG	23 (7.7)	5 (6.9)	0.8955		
Recessive	AA + AG	298 (99.7)	72 (100)	1	—	1
	GG	1 (0.3)	0 (0)	0		
Overdominant	AA + GG	277 (92.6)	67 (93.1)	1	0.34–2.57	0.9053
	AG	22 (7.4)	5 (6.9)	0.9396		

	rs4986791					
Dominant	CC	276 (92.3)	68 (94.4)	1	0.24–2.11	0.9149
	CT + TT	23 (7.7)	4 (5.6)	0.7059		
Recessive	CC + CT	298 (99.7)	72 (100)	1	—	1
	TT	1 (0.3)	0 (0)	0		
Overdominant	CC + TT	277 (92.6)	68 (94.4)	1	0.25–2.22	0.9153
	CT	22 (7.4)	4 (5.6)	0.7406		
